# Clinical characteristics and prognostic marker for hospitalization in children with influenza infection in an emergency setting

**DOI:** 10.1186/s12887-024-04882-0

**Published:** 2024-06-19

**Authors:** Rattapon Uppala, Nattapon Seenoikhao, Phanthila Sitthikarnkha, Sirapoom Niamsanit, Suchaorn Saengnipanthkul, Leelawadee Techasatian, Prapassara Sirikarn

**Affiliations:** 1 Department of Pediatrics, Faculty of Medicine, Khon Kaen University, Khon Kaen, Thailand; 2 Department of Epidemiology and Biostatistics, Faculty of Public Health, Khon Kaen University, Khon Kaen, Thailand

**Keywords:** Influenza, Children, Hospital admission, Prognostic marker, Risk factor

## Abstract

**Background:**

Influenza is a main cause of illnesses during seasonal outbreaks. Identifying children with influenza who may need hospitalization may lead to better influenza outcomes.

**Objective:**

To identify factors associated with the severity of influenza infection, specifically among children who were admitted to the hospital after being diagnosed with influenza at the emergency department.

**Methods:**

A retrospective cohort study was conducted among pediatric patients (age < 18 years) with a positive influenza rapid test who visited the emergency department at Srinagarind hospital between January2015-December2019. The dependent variable was hospital admission, while the independent variables included clinical parameters, laboratory results, and emergency severity index(ESI). The association between these variables and hospital admission was analyzed.

**Results:**

There were 542 cases of influenza included in the study. The mean age was 7.50 ± 4.52 years. Males accounted for 52.4% of the cases. A total of 190(35.05%) patients, needed hospitalization. Patients with pneumonia, those who required hospitalization or were admitted to the critical care unit, consistently exhibited an elevated absolute monocyte count and a reduced lymphocyte-to-monocyte ratio (LMR). Various factors contribute to an increased risk for hospitalization, including ESI level 1–2, co-morbidity in patients, age < 1 year old, and an LMR below 2.

**Conclusions:**

ESI level 1–2 and co-morbidity in patients represent significant risk factors that contribute to higher hospitalization admissions. A LMR below 2 can be used as a prognostic marker for hospitalization in children with influenza infection.

## Introduction

Influenza remains to be a serious concern in the context of public health, characterized by unanticipated outbreaks and pandemics [1, 2]. This is particularly evident in the pediatric population, where it leads to substantial morbidity and mortality on an annual basis [3]. Influenza infection results in a diverse array of clinical symptoms, the most of which are not severe and may be managed on an outpatient basis. However, particular children, especially those with pre-existing chronic medical conditions, might require hospitalization. Hence, there is a pressing need to implement measures aimed at reducing the morbidity and fatality rates related with influenza, particularly among the pediatric population [4–6]. Identifying children with influenza who may need hospitalization may lead to better influenza outcomes. Several clinical findings, such as elevated respiratory and heart rate, can assist physicians in identifying and prognosticating the extent of influenza infection in pediatric patients [7, 8]. Laboratory investigations, such as the complete blood count, are frequently used to assess and define the severity of infections. However, no distinct laboratory pattern has been shown to be specifically linked to the severity of influenza [9, 10]. The objective of this study was to identify factors associated with the severity of influenza infection, especially among children who were admitted to the hospital after being diagnosed with influenza in the emergency department.

## Methods

The authors conducted a retrospective cohort study from January 2015 and December 2019 by collecting the data from the Health Object Program®, an authorized electronic medical records program, at the Srinagarind Hospital, Faculty of Medicine, Khon Kaen University, Thailand. All pediatric patients under the age of 18 who tested positive for influenza using a rapid influenza test (Quicknavi-flu) and encountered in the emergency department were included in the study. Patients above the age of 18, with a non-confirmed influenza test, or who did not have a blood test were excluded from the study.

Routine complete blood count (CBC) was conducted for all patients as a routine laboratory procedure at the emergency department. The laboratory findings encompassed the total count of white blood cells (WBC) as well as the differential count of neutrophils, lymphocytes, and monocytes, together with the platelet count. The lymphocyte/monocyte ratio (LMR) was determined by dividing the total number of lymphocytes by the total number of monocytes. The CBC results were conducted and regularly documented in the HealthObject®, an electronic medical record platform utilized by the Faculty of Medicine at Khon Kaen University. The study retrieved recorded data and examined clinical characteristics such as age, gender, and co-morbidity in order to determine possible risk factors for hospitalization in influenza patients. The CBC results were obtained from the central laboratory of the university hospital for our research. This laboratory has accreditation from the Bureau of Laboratories Quality Standards and complies with ISO 15189:2012.

The hospital admission and the presence of pneumonia (defined as individuals who had a chest x-ray that was confirmed by pediatric pulmonologists) were the dependent variables, whereas the independent variables consisted of clinical characteristics (age, gender, and co-morbidity), laboratory findings (CBC and the differential counts), and emergency severity index (ESI). The ESI levels were categorized into five categories: (1) resuscitation, (2) emergency, (3) urgency, (4) lesser urgency, and (5) non-urgency.

Descriptive statistical methods, means, standard deviations (SDs), medians and frequencies were used to analyze the demographic data. The Mann-Whitney U test was performed to determine differences between two independent groups. Values of *P* < 0.05 were considered to indicate statistical significance. Multinomial logistic regression analysis was used to examine the association between proposed factors and the requirement for hospital admission.

The study was approved by the institutional review board of the Khon Kaen University, Human Research Ethics Committee (#HE641337). The need for written informed consent was waived by the Khon Kaen Universityethics committee due to the retrospective nature of the study, IRB No. HE641337.

### Statistical analysis

At the end of the study, the collected data were analyzed using STATA software version 10 (StataCorp LP). Descriptive statistical methods, means, standard deviations (SDs), medians and frequencies were used to analyze the demographic data. The Mann-Whitney U test was performed to determine differences between two independent groups. Values of *P* < 0.05 were considered to indicate statistical significance. Multinomial logistic regression analysis was used to examine the association between proposed factors and the requirement for hospital admission.

## Results

**Fig. 1:**
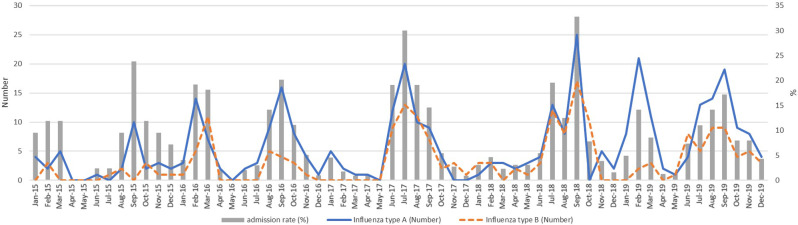
The number of confirmed cases of diagnosed influenza in children and admission rate for each following month of the year

During the study period, there were a total of 577 confirmed cases of pediatric influenza infection. Out of these cases, 35 children were eliminated. The exclusion was due to 29 cases of missing clinical data and 6 cases of missing laboratory findings obtained from the prior institution. After excluding cases, there were a total of 542 cases that were included in the study. There were 284 boys (52.4%) and 258 girls (47.6%). The mean age was 7.50 ± 4.52 years. The patients were categorized into three age groups: infants (under 1 year), 1 to under 5 years, 5 to under 12, and 12 to under 18 years. The highest frequency was observed among those aged above 5 years to under 12 years, with a total of 231 cases (42.62%). Out of the 542 individuals included in the study, 347 (64.02%) were identified with influenza A infection, whereas the remaining 195 (35.98%) had influenza B infection (Fig. 1).

**Fig. 2:**
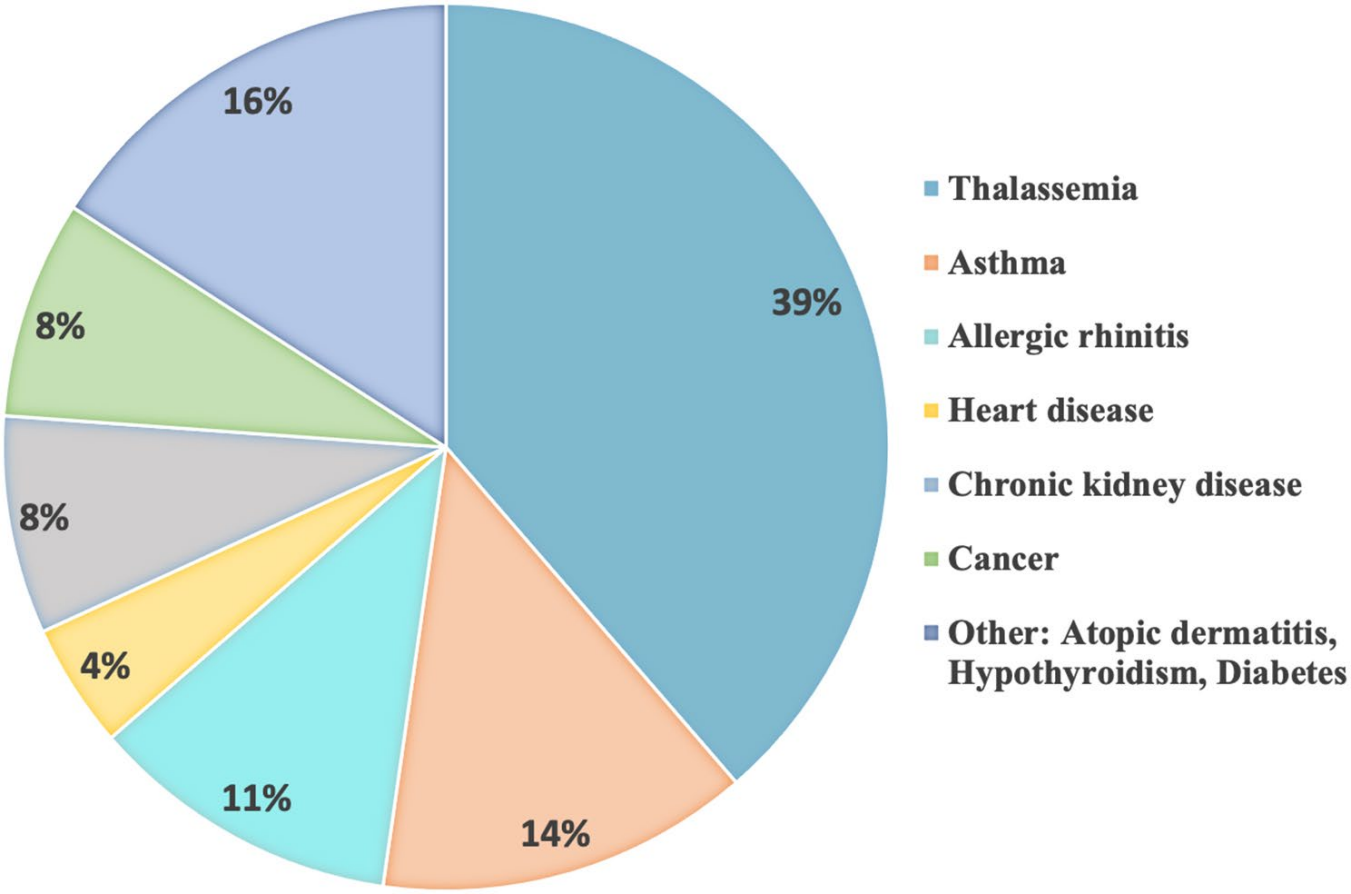
The comorbidity that was seen in the study population

A total of one hundred and ninety patients, accounting for 35.05% of the total, needed to be admitted to the hospital. Among those who required hospital admission, 29 patients were admitted in the intensive care unit (ICU). Out of the individuals who needed to be hospitalized, 71 cases (37.37%) were patients with comorbidity. Thalassemia was the most frequent comorbidity observed in the study population. The majority of thalassemia cases were of the minor type (trait-based). There was one case of Beta thalassemia, the major type, which resulted in fatality. Figure 2 depicts the comorbidity that was seen in the study population. Patients with pre-existing co-morbidities had a significantly greater risk of hospital admission compared to those without any co-morbidity, p-value < 0.001 (Table 1).

**Table 1:** Distinguishing features comparing the hospital admission and non-admission group and multi-logistic regression analysis among variables associated with hospital admission

Characteristics	Admitted (*n* = 190)				Not admitted (*n* = 352)			*P* value
**Age**								0.001
< 1 year	12 (6.32)			13 (3.69)				
1-<5 years	68 (35.79)			78 (22.16)				
5-<12 years	82 (43.16)			149 (42.33)				
12-<18 years	28 (14.73)			112 (31.82)				
**ESI level**								< 0.001
Level 1–2 (Resuscitation/Emergency)	60 (31.58)			43 (12.22)				
Level 3–5 (Urgency/Less Urgency/NonUrgency)	130 (68.42)			309 (87.78)				
**Laboratory profile from complete blood count**								
WBC count 10^9^/L, median (IQR)	7.36 (5.35–9.80)			7.04 (5.25–9.22)				0.222
Neutrophil 10^9^/L, median (IQR)	5.04 (3.02–7.23)			4.45 (2.99–6.35)				0.108
Lymphocyte (10^9^/L) 10^9^/L, median (IQR)	1.38 (0.80–2.43)			1.52 (0.91–2.30)				0.365
Monocyte 10^9^/L, median (IQR)	0.68 (0.42–1.11)			0.61 (0.42–0.81)				0.006
Platelet 10^9^/L, median (IQR)	232.00 (193.00-288.00)			237.50 (191.50–306.00)				0.349
Neutrophil-Lymphocytes ratio, median (IQR)	3.45 (1.53–7.21)			2.95 (1.60–5.71)				0.272
Lymphocytes-monocyte ratio, median (IQR)	2.02 (1.08–3.84)			2.48 (1.53–4.26)				0.004
**Multi-logistic regression analysis among variables associated with hospital admission**								
**Variables**	**Admit** **(** *n* **= 190)**	**No admit** **(** *n* **= 352)**	**Crude** **Odds Ratio (95%CI)**			**P value**	**Adjusted** **Odds Ratio (95%CI)**	**P value**
**Gender**								
Male	111 (58.42)	173 (49.15)	1			0.0388	**-**	**-**
Female	79 (41.58)	179 (50.85)	0.69 (0.48–0.98)				**-**	**-**
**Age**								
< 1 year	12 (6.32)	13 (3.69)	1			0.0006	**-**	**-**
1-<5 years	68 (35.79)	78 (22.16)	0.94 (0.40–2.21)				**-**	**-**
5-<18 years	110 (57.89)	261 (74.15)	0.46 (0.20–1.03)				**-**	**-**
**ESI level**								
Level 1–2 (Resuscitation/ Emergency)	60 (31.58)	43 (12.22)	2.61 (1.59–4.30)			< 0.001	2.63 (1.60–4.33)	< 0.001
Level 3–5 (Urgency/Less Urgency/ NonUrgency)	130 (68.42)	309 (87.78)	1				1	
**Monocyte count**								
>/= 800	76 (40.00)	89 (25.28)	1.97 (1.35–2.87)			0.0004	1.35 **(**0.85–2.13**)**	0.202
< 800	114 (60.00)	263 (74.72)	1				1	
**Lymphocytes/monocyte ratio**								
>/= 2	95 (50.00)	214 (60.80)	0.64 (0.45–0.92)			0.0156	0.63 (0.41–0.98)	0.040
< 2	95 (50.00)	138 (39.20)	1				1	
**Co-morbidity**								
Yes	71 (37.37)	17 (4.83)	11.76 (6.66–20.77)			0.040	6.50 (3.72–11.35)	< 0.001
No	119 (62.63)	335 (95.17)	1					

The ESI levels were categorized into five categories: resuscitation, emergency, urgency, lesser urgency, and non-urgency. The urgency level (ESI level 3) encompassed the majority of the study population, at 67.90%. Patients classified with ESI level 1–2 (indicating a need for immediate medical attention) had a significantly greater frequency of hospitalization compared to those with ESI level 3–5, (p-value < 0.001) (Table 1).

There was no substantial difference in the overall white blood cell count (WBC) between the group of patients who were admitted and the group who were not admitted. The study found a significant difference in the monocyte count and lymphocyte/monocyte ratio (LMR) between the admission group and the non-admission group. The admission group had a higher monocyte count and a lower LMR compared to the non-admission group, with p-values of 0.006 and 0.004, respectively, Table 1. Patients who required ICU admissions had a higher monocyte count and a lower LMR than those who were admitted to the general ward, with p-values of 0.035 and 0.006, respectively (Table 2).

**Table 2:** A comparison of the monocyte count and lymphocyte-monocyte ratio in individuals with influenza infection across various patient groups

	Influenza infection patients (*N*)		*P*-value
	Pneumonia (*n* = 84)	Non-pneumonia (*n* = 458)	
**Monocyte count** 10^9^/L, median (IQR)	0.87 (0.53–1.47)	0.60 (0.40–0.82)	< 0.001
**Lymphocytes-Monocyte ratio** median (IQR)	1.65 (0.97-3.00)	2.52 (1.49–4.48)	< 0.001
	**Admission** **(** *n* **= 190)**	**No admission** **(** *n* **= 352)**	
**Monocyte count** 10^9^/L, median (IQR)	0.68 (0.42–1.11)	0.61 (0.42–0.81)	0.006
**Lymphocytes-Monocyte ratio** median (IQR)	2.02 (1.08–3.84)	2.48 (1.53–4.26)	0.004
	**ICU** **(** *n* **= 29)**	**General ward** **(** *n* **= 161)**	
**Monocyte count** median (IQR)	0.86 (0.47–1.44)	0.65 (0.41–1.02)	0.035
**Lymphocytes-monocyte ratio** median (IQR)	1.56 (0.76–2.47)	2.28 (1.18-4.00)	0.006

Pneumonia was defined as individuals who had a chest x-ray that was confirmed by pediatric pulmonologists. In the present study, a total of eighty-four people were identified as having pneumonia. In comparison to those without pneumonia, patients with pneumonia had higher monocyte levels and lower LMR. The statistical analysis revealed p-values of less than 0.001 for both comparisons (Table 2).

The risk of hospitalization is increased by several variables, including the presence of ESI level 1–2, co-morbidity in patients, age less than 1 year old, monocyte above 0.8 × 10^9^ cells/L, and an LMR less than 2, as shown in Table 2. The multi-logistic regression analysis revealed that the presence of ESI level 1–2 (adjusted OR 2.63, *P* < 0.001), co-morbidity in patients (adjusted OR 6.50, *P* < 0.001), and LMR below 2 (adjusted OR 0.63, *P* < 0.04) were significant factors that associated with increased risk of hospitalization in influenza patients, Table 2.

Out of the study population, 99.45% were observed to have made a complete recovery and were able to be discharged from the hospital, however, three dead cases was recorded. Every mortality instance included individuals with preexisting conditions. Among the patients, there was a 6-year-old girl with preexisting Beta thalassemia, a 6-year-old boy with chronic renal disease, and a 13-year-old girl with systemic lupus erythematosus. Despite receiving oseltamivir for influenza treatment, these patients also received broad-spectrum antibiotics. Their clinical status deteriorated throughout their stay as a result of subsequent bacterial infections. Mechanical ventilation and inotropic medications were administered. Eventually, all three individuals died from multiorgan dysfunction.

## Discussion

Influenza continues to be a significant issue in public health, marked by unexpected surges and global outbreaks. This is especially noticeable in children since the disease is very infectious and spreads easily through respiratory droplets. Children are at a higher risk of acquiring the virus in environments such as schools, daycares, and playgroups [2, 3, 11]. The present study found that individuals above the age of five and less than twelve had the highest prevalence of influenza infection. This might be explained by the fact that school-aged children are more susceptible to viral exposure in crowded contexts like schools, which increases the chance of viral infection.

Based on a previous study that documented hospital admissions for influenza nationwide, there are two distinct peaks in influenza incidence in Thailand: one occurring from January to April and another from May to October [1]. However, Influenza infection is a particular strain that can occur throughout the year in Thailand. Figure 1 displays the number of confirmed cases of diagnosed influenza and admission rate in children for each following month of the year.

The Quicknavi-flu has been routinely used in emergency departments as a diagnostic tool for the identification of influenza patients in the present study. This rapid influenza kit demonstrated a sensitivity of 63% and a specificity of 96% [1]. The cost-effectiveness and efficacy of rapid influenza testing have been demonstrated in the timely acquisition of results during emergency situations. In our institution, PCR has been implemented selectively in emergency situations, as opposed to being implemented universally.

Despite the fact that influenza infections were uncommonly fatal, they did occur. The present study identified three instances of mortality and 29 admissions to the intensive care unit (ICU). Consequently, it is imperative to identify predictive factors for severe influenza. A previous publication revealed the yield of higher respiratory rate adjusted for age was the most useful vital sign predictor of severity among young children with PCR-confirmed influenza [8]. The present study revealed the yield of ESI level at the emergency setting. In comparison to influenza patients with an ESI level of 3–5, those with an ESI level of 1–2 were 2.63 times more likely to require hospital admission (95% CI 1.60–4.33, *P* < 0.001). Among ESI levels 3–5, ESI level 3 (urgency) had the greatest number of hospital admissions (129 out of 130), with only one case at ESI level 4 (lesser urgency) and none at ESI level 5 (non-urgency). This discovery provides the benefit for the use of ESI as a triage tool in an emergency setting. The ESI, a triage algorithm with five levels, was designed to distribute medical care and offer suitable medical assistance to patients with minimal complexity who fall into levels 4 and 5. Those in this category often consume fewer resources and, in rare cases, need hospitalization [13]. 

In the present study, 190 patients (35.05%) were hospitalized. The number of hospitalizations is comparatively high [1]. This might be attributed to the fact that the study was carried out at a highly specialized hospital where the cases tended to be more severe. This could account for the higher rate of hospitalization compared to other hospitals.

To identify laboratory indicators in influenza severity prediction, hematological profiles regularly conducted in an emergency room are explored to determine potential associations. Although some specific cytokine profiles such as high Th1, low Th2, high IL-6, and low Th17 cytokines may serve as reliable indicators and independent risk factors for intermittent positive pressure ventilation in patients with influenza infection [14]. Nevertheless, these specific cytokines are hardly investigated within routine healthcare settings. Therefore, a regular hematological profile may provide more advantages if it aids in forecasting the severity of influenza infection. Previous studies have found that patients with acute influenza A infection displayed increased intermediate monocytes frequencies in blood [15, 16]. Another study undertaken in an emergency setting has discovered that peripheral monocytosis, which is defined as more than 0.8 × 10^9^ cells/L, can be a predictor factor for unfavorable outcomes in the emergency department [17]. The present study also showed that influenza patients with pneumonia (diagnosed as those who had relevant chest x-ray confirmed by pediatric pulmonologists) had a significantly higher absolute monocyte count than those without pneumonia (P-Value < 0.001). Additionally, individuals with influenza who necessitated hospitalization and those who required ICU admission exhibited elevated absolute monocyte counts in comparison to those who were admitted to the general ward and did not require hospitalization. (Table 2)

LMR was another key indicator demonstrating a lower ratio in the pneumonia group. Both absolute monocytes count and LMR showed a comparable pattern in characterizing the severity of influenza infection in patients requiring hospitalization and those admitted to the intensive care unit, Table 2.

A low LMR, particularly below 2, has been considered as an indication for identifying influenza infection [18, 19]. The present study discovered that a decreased LMR can also be detected in influenza infections that need hospitalization. When a particular cut-off LMR below 2 was used in the study population, influenza patients who required hospitalization had a considerably higher incidence of a cut-off LMR below 2.

The presented study is subject to certain limitations as a result of the retrospective study design. We did not get immunization data through record review, which may explain some variations in the severity of influenza condition. Some crucial features such as the vital signs and other relevant clinical information were not included in the study due to an inability to collect adequate data from medical records.

## Conclusions

The ESI level serves as a valuable screening tool for determining the need for hospital admission. An increase in the number of monocytes in the blood and a low lymphocyte-to-monocyte ratio (LMR) are frequently observed in patients with severe influenza who need to be hospitalized or admitted to the intensive care unit. A low LMR of less than 2 can serve as a predictive indicator for hospitalization in children with influenza infection.

## Data Availability

Data availability upon request with corresponding author.
